# Characterization of Wnt and Notch-Responsive Lgr5+ Hair Cell Progenitors in the Striolar Region of the Neonatal Mouse Utricle

**DOI:** 10.3389/fnmol.2018.00137

**Published:** 2018-04-30

**Authors:** Dan You, Luo Guo, Wenyan Li, Shan Sun, Yan Chen, Renjie Chai, Huawei Li

**Affiliations:** ^1^ENT Institute and Otorhinolaryngology, Department of Affiliated Eye and ENT Hospital, Key Laboratory of Hearing Medicine of NHFPC, Shanghai Engineering Research Centre of Cochlear Implant, State Key Laboratory of Medical Neurobiology, Fudan University, Shanghai, China; ^2^Key Laboratory for Developmental Genes and Human Disease, Ministry of Education, Jiangsu Province High-Tech Key Laboratory for Bio-Medical Research, Institute of Life Sciences, Southeast University, Nanjing, China; ^3^Co-innovation Center of Neuroregeneration, Nantong University, Nantong, China; ^4^Institutes of Biomedical Sciences and The Institutes of Brain Science and the Collaborative Innovation Center for Brain Science, Fudan University, Shanghai, China

**Keywords:** supporting cells, stem cell, regeneration, utricle, hearing and balance

## Abstract

Dysfunctions in hearing and balance are largely connected with hair cell (HC) loss. Although regeneration of HCs in the adult cochlea does not occur, there is still limited capacity for HC regeneration in the mammalian utricle from a distinct population of supporting cells (SCs). In response to HC damage, these Lgr5+ SCs, especially those in the striolar region, can regenerate HCs. In this study, we isolated Lgr5+ SCs and Plp1+ SCs (which originate from the striolar and extrastriolar regions, respectively) from transgenic mice by flow cytometry so as to compare the properties of these two subsets of SCs. We found that the Lgr5+ progenitors had greater proliferation and HC regeneration ability than the Plp1+ SCs and that the Lgr5+ progenitors responded more strongly to Wnt and Notch signaling than Plp1+ SCs. We then compared the gene expression profiles of the two populations by RNA-Seq and identified several genes that were significantly differentially expressed between the two populations, including genes involved in the cell cycle, transcription and cell signaling pathways. Targeting these genes and pathways might be a potential way to activate HC regeneration.

## Introduction

Balance in mammals is maintained by the activity in a subdivision of the inner ear known as the vestibular system, and the primary organ involved in balance is the utricle. The sensory epithelium of the utricle is composed of hair cells (HCs) and supporting cells (SCs) in the basal layer (Huch et al., 2013), and it has two anatomical zones—a central striolar region and a surrounding extrastriolar region (Desai et al., 2005). The striolar region is a crescent-shaped region, and the specialized afferent neuron terminals are located in this region (Burns and Stone, 2017).

Forge identified a large number of cells with immature hair bundles in multiple stages of development in the utricle after gentamicin-induced HC death (Forge et al., 1993). Recently, several studies have shown that new HC regeneration can occur after HC damage to restore the function of the utricle (Li et al., 2003; Lin et al., 2011; Wang et al., 2015), and increasing evidence suggests that utricular SCs serve as a reliable source to partially regenerate HCs either via direct trans-differentiation or by mitotic regeneration (Sinkkonen et al., 2011). For the purpose of restoring full utricular function, several studies have made efforts to promote SC proliferation and HC regeneration (Lin et al., 2011; Burns et al., 2012b), and identifying genes that regulate the proliferation and HC regeneration ability of SCs is very important for developing new therapeutic strategies for HC regeneration.

The utricular SCs appear to be quite homogeneous; however, analyzing the results of many previous studies shows that regenerated HCs are consistently more concentrated in the striolar region than the extrastriolar region of the mammalian utricle (Lin et al., 2011; Wang et al., 2015; Gao et al., 2016). However, a detailed analysis of the differences between the striolar and extrastriolar SCs has not yet been performed.

*Lgr5*, a downstream target gene of the Wnt signaling pathway, acts as a stem cell marker in multiple organs, including the small intestine, liver, pancreas, and inner ear (Barker et al., 2007; Chai et al., 2012; Shi et al., 2012; Huch et al., 2013). Our previous study in the neonatal mouse utricle showed that Lgr5 is undetectable in the vestibular epithelia, but it can be reactivated specifically in the striolar SCs after neomycin injury (Wang et al., 2015). The *Plp1* gene was first found in the nervous system because it encodes proteolipid protein (PLP), which is one of the most important proteins in the myelin sheath (Doerflinger et al., 2003). Morris et al. (2006) and Gómez-Casati et al. (2010) reported that the *Plp1* promoter is also active in SCs of the inner ear during both embryonic stages and postnatal ages. Burns et al. (2012b) discovered that the *Plp1* promoter is normally active in most of the SCs that reside within the extrastriolar region of the mouse utricle and that the expression level declines with age.

In this study, we used flow cytometry to isolate the Lgr5+ SCs and Plp1+ SCs from transgenic mice so as to characterize the differences between the two subsets of SCs in terms of proliferation, differentiation, and response to signaling pathways. Furthermore, we performed RNA-Seq to identify the differentially expressed genes that might lead to the differences between striolar and extrastriolar SCs.

## Materials and Methods

### Experimental Animals

Wild-type C57BL/6J mice were purchased from Fudan Medical School (Shanghai, China). Lgr5-EGFP-IRES-creERT2 mice (Stock#008875), Plp1-CreERT (Jackson Laboratory, Cat. #5975), and Rosa26R-tdTomato (Jackson Laboratory, Cat. #7908) mice in the C57BL/6J background were purchased from the Jackson Laboratory. Plp1-creERT mice (heterozygous) were crossed with Rosa26R-tdTomato mice (homozygous) to trace the Plp1+ cell fate in the utricles. All of the genotyping primers are listed in Supplementary Table S2.

For Cre activation, tamoxifen was injected into P1 mice. All animal procedures were approved by the Animal Care and Use Committee of Fudan University and were consistent with the National Institutes of Health Guide for the Care and Use of Laboratory Animals. All efforts were made to minimize the number of animals used and to prevent their suffering.

### Utricle Explant Culture

The mice were euthanized at P2, and the utricles were isolated from the temporal bone in tissue culture medium under sterile conditions. The otoconia in the utricles were gently removed with fine forceps. Whole organs were cultured in DMEM/F12 (Invitrogen) supplemented with 1% N2 (Invitrogen), 2% B27 (Invitrogen), and ampicillin (50 μg/ml; Sigma-Aldrich) at 37°C with 5% CO_2_ in 4-well Petri dishes (Greiner Bio-one). Neomycin sulfate (1 mM, Sigma-Aldrich) was added to kill the HCs. The explanted utricles were treated with 5 μM 6-Bromoindirubin-3′-oxime (BIO)-Acetoxime (Sigma) or 50 μM N-[N-(3,5-difluorophenacetyl)-l-alanyl]-S-phenylglycine t-butyl ester (DAPT; a γ-secretase inhibitor IX, EMD (DAPT). Dimethyl sulfoxide (DMSO; 0.5%; Sigma Aldrich) was used for the negative control. To label proliferating cells, 10 μM 5′-ethynyl-2′-deoxyuridine (EdU; Life Technologies) was added to the culture media for the entire culture period.

### Cell Sorting by Flow Cytometry

Utricles from Lgr5-EGFP-CreERT2+ and Plp1-tdTomato+ mice were cultured with 1 mM neomycin for 24 h to damage the HCs and to re-induce the expression of Lgr5. To obtain a single-cell suspension, all tissues were gently removed from the Petri dishes (Greiner Bio-one) using fine forceps and subjected to 0.125% trypsin at 37°C for 10 min. Soybean trypsin inhibitor was added to stop the enzymatic reaction. The cell suspension was carefully triturated with plastic pipette tips (epTIPS Filter 20**–**300 μl; Eppendorf), and the cells were passed through a 40 μm cell filter (Greiner Bio-one) and collected in an Eppendorf tube. Dissociated cells were sorted on a MoFlo^®^ SX fluorescence activated cell sorting (FACS) cytometer (Beckman Coulter, CA, USA) using the channels for GFP and tdTomato, and the positive fractions were collected for further experiments.

### Immunohistochemistry and Image Acquisition

After fixing for 30 min at room temperature with 4% paraformaldehyde (Sigma-Aldrich) in 0.1 M phosphate buffer, the utricles were blocked with 10% donkey serum in 1% PBST (1% Triton-X100 in 10 mm PBS) at pH 7.4 for 1 h at 37°C. Tissues were rinsed with PBS and then incubated with primary antibodies (diluted with 1% PBST) for 1 h at 37°C and then at 4°C overnight. The primary antibodies were rabbit anti-Myosin7a (1:500 dilution; Proteus Biosciences), goat anti-Sox2 (1:300 dilution, Santa Cruz Biotechnology), and chicken anti-EGFP (1:800 dilution; Abcam). After being washed with PBS on the following day to remove the unbound antibodies, the specimens were incubated with the appropriate Alexa-conjugated secondary antibodies (diluted in 1% PBST) for detection at 4°C overnight. Appropriate Alexa-conjugated secondary antibodies were used for detection. Proliferative cells were labeled with EdU (Click-iT EdU Imaging kit, Life Technologies) for 40 min at room temperature according to the manufacturer’s protocol. DAPI (1:800 dilution; Sigma-Aldrich) was used to show the cell nuclei. Tissues were mounted in anti-fade fluorescence mounting medium and coverslipped. Fluorescent images were acquired using a Leica SP8 confocal microscope. All images were processed using ImageJ and Adobe Photoshop CC.

### Genotyping and Quantitative RT-PCR

Standard PCR was used for transgenic mouse genotyping using genomic DNA isolated from mouse tail tips, and quantitative real-time polymerase chain reaction (qRT-PCR) was performed to determine gene expression levels. Total RNA of the sorted cells was isolated using the RNeasy micro kit (QIAGEN), and mRNA was reverse transcribed with the PrimeScript™ II 1st Strand cDNA Synthesis Kit (Takara, Japan). qRT-PCR reactions were performed with SYBR Premix ExTaq II (Takara) on a 7500HT Fast Real-Time PCR System (Applied Biosystems, Foster City, CA, USA). Each PCR reaction was carried out in triplicate. With GAPDH as the endogenous reference, the relative quantification of gene expression was analyzed using the ΔΔCT method. Primer pairs for the qPCR were designed with the Primer3 online software. All of the primers are listed in Supplementary Table S3.

### Sphere-Forming Assay and Differentiation Assay

After being sorted, Lgr5+ and Plp1+ cells were plated separately in 12-well ultra-low-attachment plates (Corning, NY, USA) at a density of 2000 cells/ml. The culture media was DMEM/F12 medium (Invitrogen) with 1% N2 and 2% B27, EGF (20 ng/ml), bFGF (10 ng/ml), IGF-1 (50 ng/ml), and heparin sulfate (50 ng/ml; all from Sigma). For activation of the Wnt signaling pathway during the sphere-forming assay, 5 μM BIO-acetoxime (Sigma) was added to the culture media. Both the sphere number and diameter were determined every 5 days. For propagation assays, the spheres that had been cultured for 5 days were collected and dissociated with 0.25% trypsin at 37°C for 6 min, and 10% serum/DMEM media was added to block the reaction. The single cells were re-cultured in non-adhesive 12-well culture clusters. The propagation assay was repeated at 5-day intervals. For cell differentiation in spheres, the colonies were plated on laminin-coated dish and cultured for 10 days in DMEM/F12. EdU (10 μM, Invitrogen) was added to the culture medium to label dividing cells.

### Culture of Flow-Sorted Cells

Purified Lgr5+ and Plp1+ cells were cultured at a density of 20 cells/μl on a laminin-coated dish using DMEM/F12 medium supplemented with 1% N2 and 2% B27, EGF (20 ng/ml), bFGF (10 ng/ml), IGF-1 (50 ng/ml), and heparin sulfate (50 ng/ml). For inhibiting the Notch signaling pathway during cell differentiation, 50 μM DAPT (a γ-secretase inhibitor IX, EMD) was added. EdU was included in the culture medium to measure cell proliferation.

### Cell Counting and Statistics

After the whole tissue culture experiments, three randomly chosen representative pictures from the striolar region and extrastriolar region were used for analysis. The numbers of HCs and SCs in the striolar region and extrastriolar region were counted using ImageJ. For all experiments, *n* values represent the numbers of organs or samples examined unless otherwise stated. Statistical analyses were conducted using GraphPad Prism 5.0 software. Two-tailed, unpaired Student’s *t*-tests were used to determine statistical significance when comparing two groups, and one-way ANOVA was used when comparing more than two groups. Furthermore, the numbers and diameters of spheres from the first five passages were analyzed by repeated-measures two-way ANOVA and the Holm–Sidak multiple comparison test. Data are shown as the mean ± SD. A value of *p* < 0.05 was considered to be statistically significant.

### RNA-Seq and Data Analysis

RNA-Seq libraries of FACS-purified cells were prepared with the SMART-Seq v4 Ultra Low Input RNA Kit for Sequencing and the Illumina mRNA-Seq Sample Prep Kit. SPRI beads (Ampure XP, Beckman) were used for size selection in each purification step after RNA fragmentation. All libraries were analyzed for quality and concentration using an Agilent Bioanalyzer and Qubit 2.0 Fluorometer, and 2× 150 bp paired-end sequences were generated on an Illumina HiSeq2500 Platform. Fastq files of paired-end reads sequences were obtained and were trimmed with Trimmomatic (Trapnell and Schatz, 2009). Clean reads were mapped to the mouse reference genome (mm10) using TopHat followed by transcript assembly and differential gene expression analysis using Cufflinks. The expression of every gene was measured by FPKM (Fragments Per Kilobase of transcript per Million fragments mapped). Genes and transcripts were annotated using gene transcription information from the RefGene database (NCBI). *P*-values were adjusted using the Benjamini and Hochberg multiple testing procedure. Genes with adjusted *p*-values < 0.01 were marked as significant. To assess the extent of functional enrichment, we performed gene ontology analysis with the functional annotation tool DAVID 6.7 (Huang da et al., 2009), which determines whether biological processes are enriched within a list of genes.

## Results

### SCs in the Striolar Region Marked by Lgr5-EGFP and in the Extrastriolar Region Marked by Plp1-tdTomato Had Different Abilities to Proliferate and Differentiate Into HCs After Neomycin Damage *in Vitro*

**Figure 1:**
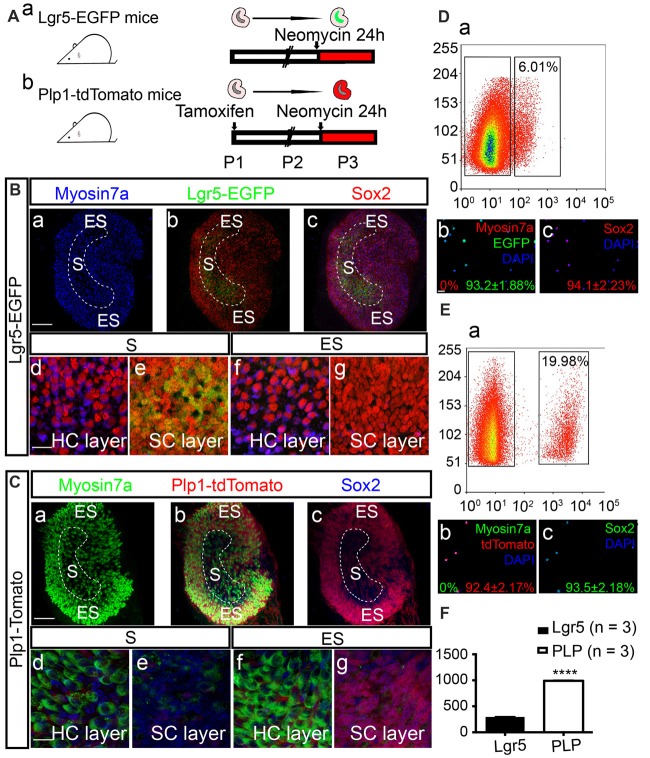
Expression patterns of Lgr5 and Plp in the utricle after neomycin damage. **(A)** Tamoxifen was injected into Plp1-CreERT-tdTomato mice on P1 to activate Cre recombinase. Utricles from both Lgr5-EGFP-CreERT mice **(Aa)** and Plp1-CreERT-tdTomato mice **(Ab)** were isolated for culture on P2. The utricles were cultured with neomycin for 24 h and then collected for analysis on P3. **(B,C)** Images showing the Lgr5-EGFP and Plp1-tdTomato expression patterns in utricles. High-magnification images show the hair cell (HC) nuclear layer and supporting cell (SC) nuclear layer of both the striolar region (S) and extrastriolar region (ES; **Bd–g,Cd–g)**. After 24 h of neomycin exposure, HC death was observed primarily in the striolar region **(Ba,Ca)**. Neomycin-induced HC damage activated SCs to re-express Lgr5-EGFP in the same region **(Bb,c,Be)**, and tdTomato mainly labeled SCs in the extrastriolar region in Plp-CreERT-tdTomato mice **(Cbc,Cg)**. **(D,E)** Lgr5-EGFP+ SCs and Plp1-tdTomato+ SCs were isolated by fluorescence activated cell sorting (FACS). The percentage of Lgr5-EGFP+ cells was 6.02% **(Da)**, and the percentage of Plp1-tdTomato+ cells was 19.98% **(Ea)**. Immunostaining confirmed that purified Lgr5+ cells robustly expressed EGFP (93.2% ± 1.88%) and Sox2 (94.1% ± 2.33%), but not the HC marker Myosin7a (0.0%; **Db,c)**. Of the isolated Plp1+ cells, 92.4% ± 1.2% were tdTomato+, 93.5% ± 2.18% were Sox2+, and none were Myosin7a+ **(Eb,c)**. **(F)** The numbers for Lgr5-EGFP+ and Plp1-tdTomato+ cells per utricle. Data are shown as the mean ± SD; *t*-test, ****p < 0.001, *n* = 3. Scale bars are 100 μm in **(Ba–c)** and **(Ca–c)** and 20 μm in **(Bd–g,Cd–g,Db,c,Eb,c)**.

Tamoxifen (0.2 mg/g) was administrated to Plp1-CreERT/+; Rosa26R-tdTomato/+ mice at postnatal day 1 (P1) to induce activation of Cre recombinase, resulting in permanent labeling (tdTomato) of Plp1+ cells. Both Plp1-tdTomato and Lgr5-EGFP mice were then sacrificed on P2, and the utricles were dissected and cultured with neomycin for 24 h (Figure 1A). Consistent with previous reports (Wang et al., 2015), we found that after neomycin damage Lgr5-EGFP specifically labeled the striolar SCs and Plp1-tdTomato mainly labeled the extrastriolar SCs in the utricular epithelium (Figures 1B,C). We then purified the cells by FACS (Figures 1D,E). We found that 6.01% of the utricular cells of Lgr5-EGFP mice were EGFP+ and 19.98% of the utricular cells of Plp1-tdTomato mice were tdTomato+. Immunostaining immediately after sorting confirmed cell purity. Of the Lgr5-EGFP+ cells, 93.2% ± 1.88% were EGFP+, 0% were Myosin7a+ (Myosin7a is the HC marker), and 94.1% ± 2.23% were Sox2+ (Sox2 is the SC marker), and of the Plp1-tdTomato+ cells 92.4% ± 2.17% were tdTomato+, 0% were Myosin7a+, and 93.5% ± 2.18% were Sox2+. Quantitative data showed that the total numbers of sorted cells from each utricle were 294.7 ± 3.28 (*n* = 3) Lgr5-EGFP+ cells and 990.3 ± 6.96 (*n* = 3) Plp1-tdTomato+ cells (*t*-test, *t* = 109.2, *p* < 0.001; Figure 1F). These data showed that we had established a reliable model that can effectively isolate the SCs in the striolar or the extrastriolar region for further analysis.

To further investigate the proliferation rate of SCs purified by FACS, we cultured the cells in low-attachment wells for 5 days to generate spheres. The spheres were then passaged for a continuous five generations. Observed under the fluorescence microscope, we found that the spheres derived from Lgr5-EGFP+ SCs gradually lost Lgr5 expression in culture; while the spheres derived from Plp1-tdTomato+ SCs permanently expressed tdTomato (Supplementary Figure S1A). The number of spheres from Lgr5-EGFP+ SCs were greater than those from Plp1-tdTomato+ SCs (two-way ANOVA: *F*_(4,30)_ = 216.6, ***p < 0.001). The sphere diameters of both groups did not change much from 1 to 5 generations (one-way ANOVA: Lgr5+, *F*_(4,45)_ = 1.354, *p* = 0.2648; Plp1-tdTomato, *F*_(4,45)_ = 2.342, *p* = 0.0692; Supplementary Figures S1B,C). To further evaluate the HC regeneration ability of the spheres derived from these two SC populations, we then isolated and cultured the spheres in the first generation for 10 days to allow them to differentiate. To mark cell proliferation, EdU was added from day 4 to day 7 during the culture, and the cells were fixed and then labeled with Myosin7a and EdU after culture. We found that almost all spheres derived from both SC populations could generate Myosin7a+ HCs. However, each sphere derived from Lgr5-EGFP+ SCs generated many more Myosin7a+ or EdU+/Myosin7a+ HCs than the spheres derived from Plp1-tdTomato+ SCs (unpaired *t*-test, *t* = 15.59, *p* < 0.001, *n* = 4, Supplementary Figures S1D–F).

To analyze the cells’ ability to regenerate HCs, we cultured 2000 cells in laminin-coated 4-well dishes at a density of 20 cells/μl for 10 days in serum-free medium, and EdU was added from day 4 to day 7. We found that the EGFP signal gradually disappeared, while the tdTomato was permanently expressed throughout the culture period. The Lgr5+ SCs generated many more total colonies and many more Myosin7a+ colonies than the Plp1+ SCs (Supplementary Figure S2, Table S1). Moreover, Lgr5+ SCs regenerated more Myosin7a+ and EdU+/Myosin7a+ HCs both inside and outside of the colonies than Plp1+ SCs. These results suggested that Lgr5+ SCs have higher cell proliferation and HC regeneration capability than Plp1+ SCs *in vitro*.

### SCs in the Striolar and Extrastriolar Regions Respond Differently to Wnt and Notch Signaling After Neomycin Damage *in Vitro*

**Figure 2:**
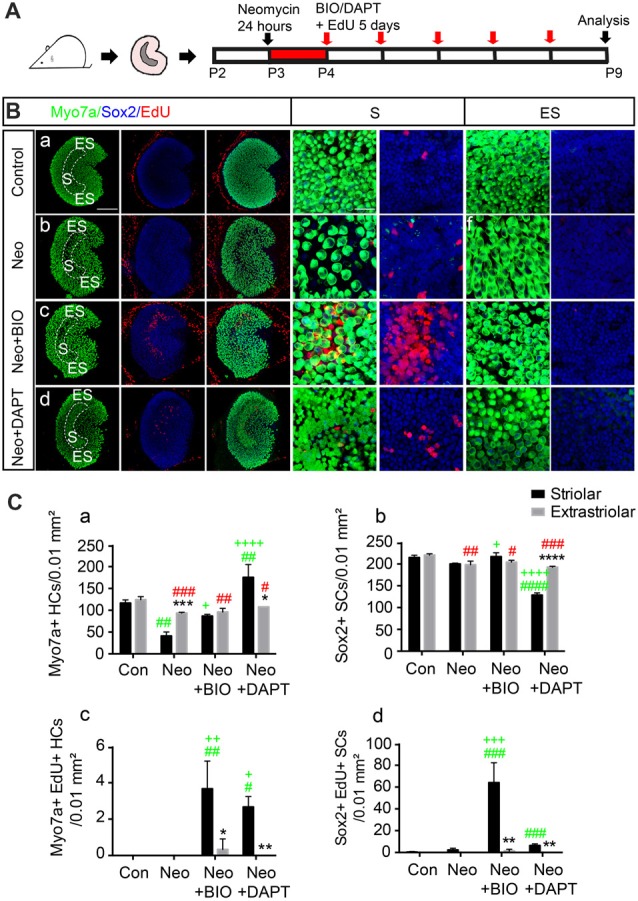
Subset of SCs in the striolar and extrastriolar region in response to Wnt and Notch signaling *in vitro*. **(A)** Schematic of the experimental procedure. **(B)** Utricles from control **(a)**, neomycin **(b)**, neomycin+6-Bromoindirubin-3′-oxime (BIO; **c**), and neomycin+N-[N-(3,5-difluorophenacetyl)-l-alanyl]-S-phenylglycine t-butyl ester (DAPT; **d**) groups were dissected and cultured. 5′-ethynyl-2′-deoxyuridine (EdU) was used to label the proliferating cells. All images are low-magnification images showing cultured utricles in the different treatment groups. High-magnification slices show the HC nuclear layer and SC nuclear layer of both the striolar and extrastriolar regions. **(C)** Quantification of Myosin7a+, Sox2+ and EdU+ cells in the striolar and extrastriolar regions in each group **(Ca–d)**. Data are shown as the mean ± SD. *p < 0.05, **p < 0.01, ***p < 0.001, ****p < 0.0001 (Striolar vs. Extrastriolar). ^#^(Green) *p* < 0.05, ^##^*p* < 0.01, ^###^*p* < 0.001, ^####^*p* < 0.0001 (Neo, Neo+BIO, Neo+DAPT vs. Con in Striolar). ^+^*p* < 0.05, ^++^*p* < 0.01, ^+++^*p* < 0.001, ^++++^*p* < 0.0001 (Neo+BIO, Neo+DAPT vs. Neo in Striolar). ^#^(Red) *p* < 0.05, ^##^*p* < 0.01, ^###^*p* < 0.001, ^####^*p* < 0.0001 (Neo, Neo+BIO, Neo+DAPT vs. Con in Extrastriolar). In **(B)**, *n* = 3. Scale bars are 100 μm in the low-magnification images and 20 μm in the high-magnification images in **(B)**.

To further investigate the response of striolar and extrastriolar SCs to Wnt and Notch signaling, we used BIO), an effective GSK3β inhibitor, as the Wnt agonist and DAPT, a γ-secretase inhibitor, as the Notch inhibitor. Utricles from P2 wild type mice were dissected and cultured with either 1 mM neomycin or vehicle for 24 h, and this was followed by 1 mM EdU with DMSO as the control or with 5 μM BIO or 50 μM DAPT to activate Wnt or inhibit Notch signaling, respectively (Figure 2A). Myosin7a+ HC, Sox2+ SC, and EdU+ proliferating cells were counted from four randomly selected extrastriolar and striolar 100 μm × 100 μm regions per specimen.

Consistent with a previous report (Burns et al., 2012a), HC density was similar in the two regions of the intact utricle (116.33 ± 7.23 and 120.67 ± 5.03 Myosin7a+ HCs per 100 μm^2^ in the striolar and extrastriolar regions, respectively, *n* = 3), and rare proliferative SCs were found only in the striolar region (1 ± 1.73 EdU+/Sox2+ SCs per 100 μm^2^, *n* = 3; Figure 2B). Robust HC loss followed neomycin damage in the striolar region compared to extrastriolar region (41.00 ± 9.00 and 93.33 ± 2.08 surviving HCs per 100 μm^2^, respectively, *n* = 3), and the number of EdU+ SCs was increased in the same field (4 ± 2 EdU+ SCs per 100 μm^2^, *n* = 3). When the Wnt signaling pathway was activated by 5 μM BIO, the numbers of both EdU+ SCs and total SCs were increased significantly in the striolar region (64.00 ± 18.33 EdU+/Sox2+ SCs and 216.67 ± 9.07 Sox2+ SCs per 100 μm^2^, respectively, *n* = 3). Interestingly, proliferating SCs mostly existed in the striolar region, while few EdU+ SCs were found in the extrastriolar region even with BIO, suggesting that only striolar SCs are responsive to Wnt signaling.

Furthermore, in the presence of DAPT, the number of HCs increased more than four-fold compared to the DMSO controls in the striolar region (175.33 ± 29.30 and 41 ± 9 HCs per 100 μm^2^, respectively, *t*-test, *t* = 7.592, *p* < 0.01, *n* = 3), while it was only slightly increased in the extrastriolar region compared to controls (107.70 ± 2.51 and 93.33 ± 2.08 HCs per 100 μm^2^, respectively, *t*-test, *t* = 7.601, *p* = 0.0016, *n* = 3; Figure 2C), which suggests that striolar SCs are more responsive to Notch signaling than extrastriolar SCs to Notch inhibition. Thus, we concluded that the SCs in these two regions responded differently to the Wnt and Notch signaling pathways.

### Spheres Derived From Lgr5+ and Plp1+ SCs Respond Differently to Wnt and Notch Signaling

**Figure 3:**
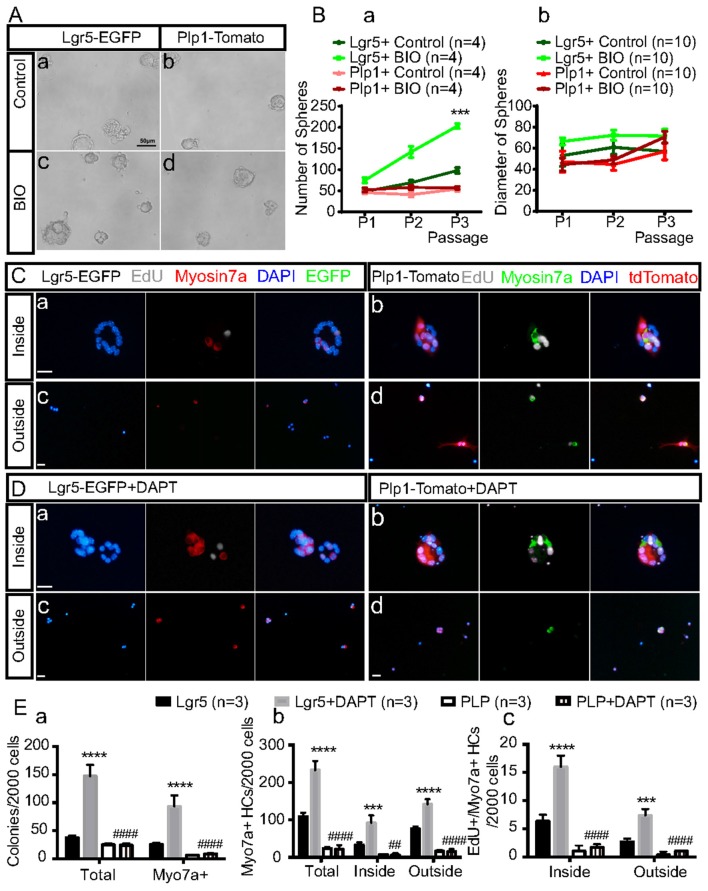
Sorted Lgr5+ SCs and Plp1+ SCs during sphere formation and HC differentiation in response to Wnt and Notch signaling *in vitro*. **(A)** Activation of the Wnt signaling pathway with BIO during neurosphere formation from the first generation in both subsets of purified SCs *in vitro* **(Ac,d)**. The control group was cultured only with media **(Aa,b)**. **(B)** Quantification of the sphere number **(Ba)** and sphere diameter **(Bb)** formed from both subsets of SCs in the BIO and control group over the first three generations ***p < 0.001 (Lgr5+Control vs. Lgr5+BIO). **(C,D)** HC differentiation assay with purified Lgr5+ **(Ca,c,Da,c)** or Plp1+ **(Cb,d,Db,d)** SCs in the presence of Notch inhibition by DAPT. The inside of the colonies represents the mitotically regenerated HCs **(Ca,b,Da,b)**, and the outside of the colonies represents the directly differentiated HCs **(Cc,d,Dc,d)**. **(E)** Quantification of total colonies, Myosin7a+ colonies and Myosin7a+ cells and Myosin7a+/EdU+ cells both inside and outside the colonies. Data are shown as the mean ± SD. ***p < 0.001, ****p < 0.0001 (Lgr5 vs. Lgr5+DAPT).^##^*p* < 0.01, ^####^*p* < 0.0001 (Lgr5+DAPT vs. PLP+DAPT). In **(Ba)**, *n* = 4. In **(Bb)**, *n* = 10. In **(E)**, *n* = 3. Scale bars are 50 μm in **(A)** and 20 μm in **(C,D)**.

To further investigate the differences in SC proliferation and differentiation between the striolar and extrastriolar regions in response to Wnt activation or Notch inhibition, purified SCs were cultured with BIO (5 μM) during the sphere-forming stage or DAPT (50 μM) during the HC differentiation stages, respectively. We found that with BIO the number of spheres derived from Lgr5+ SCs increased by 1.56 ± 0.13, 2.06 ± 0.05, and 2.08 ± 0.20-fold compared to the DMSO control in the first, second, and third generations, respectively, and the diameters of the spheres also increased compared to controls (Figures 3A,B).

With DAPT, the total colony number and Myosin7a+ colony number increased 3.96 ± 0.79 and 3.77 ± 1.18 fold, respectively, compared to the DMSO control for the Lgr5+ SC group, while the numbers of total colonies and Myosin7a+ colonies were only slightly increased after Notch inhibition in the Plp1+ SCs. In addition, with DAPT the total numbers of Myosin7a+ HCs and EdU+/Myosin7a+ HCs were increased by 2.87 ± 0.64 and 1.86 ± 0.18-fold, respectively, inside the colonies and by 2.55 ± 0.26 and 2.89 ± 1.02-fold, respectively, outside the colonies compared to DMSO controls for the Lgr5+ SC-derived spheres while only 1 or 2 newly generated HCs were seen after Notch inhibition in the Plp+ SC-derived spheres (Figures 3C–E). These results indicated that Lgr5+ SC-derived spheres were more responsive to both Wnt activation and Notch inhibition than Plp1+ SCs in terms of HC differentiation.

### RNA-Seq Analysis and Differentially Expressed Genes in Lgr5+ SCs and Plp1+ SCs

**Figure 4:**
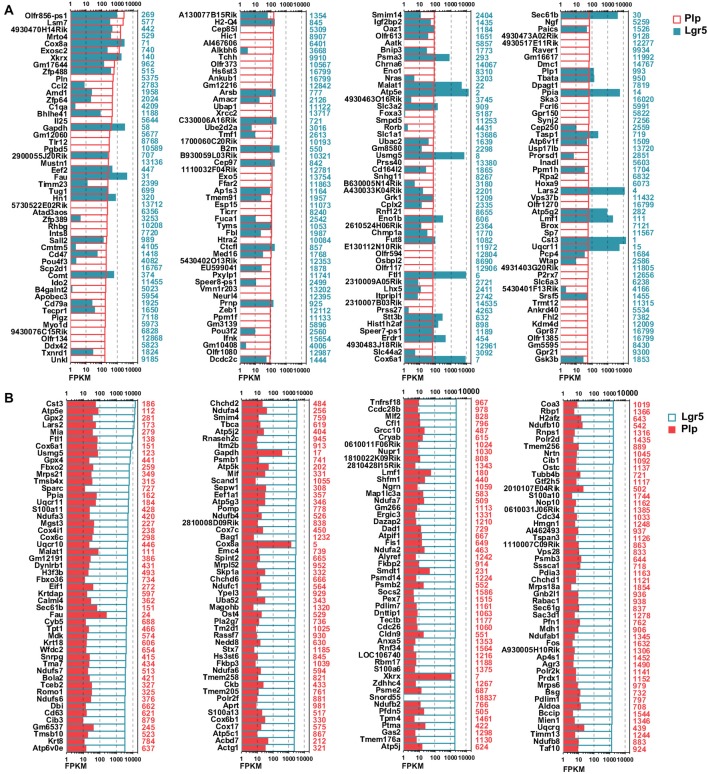
Top expressed genes in Lgr5+ and Plp1+ SCs. **(A)** The expression levels of the top 200 genes in Plp1+ SCs in descending order. The length of the bar after each gene represents its Fragments Per Kilobase of transcript per Million fragments mapped (FPKM) value. The numbers on the right side of each panel represent the rankings of the same genes in Lgr5+ SCs. **(B)** The expression levels of the top 200 genes in Lgr5+ SCs in descending order. The length of the bar after each gene represents its FPKM value. The numbers on the right side of each panel represent the rankings of the same genes in Plp1+ SCs.

To investigate the detailed gene expression profile differences between Lgr5+ striolar SCs and the other Plp1+ extrastriolar SCs, we performed RNA-Seq analysis. Three biological replicates were generated for Lgr5+ striolar SCs and Plp1+ extrastriolar SCs, respectively. Between 41.2 and 54.3 million paired-end reads were obtained for each sample, with 48.5%–89.0% of the read pairs mapping correctly to the reference genome (Mouse mm10). The expression of every gene was measured by FPKM (Fragments Per Kilobase of transcript per Million fragments mapped). We ranked the genes in each condition from large to small by their FPKM value, and the most highly expressed genes in both populations are shown in Figure 4. For comparison, the expression levels of both groups for the same transcripts (color bar) as well as the abundance rankings for these transcripts (colored numbers beside each panel) are given on the right side of the figure. Figure 4A shows the expression levels for the top 200 most abundant transcripts in Plp1+ extrastriolar SCs compared to the same transcripts in Lgr5+ striolar SCs. Figure 4B similarly shows the 200 most abundant transcripts in Lgr5+ striolar SCs compared to the same transcripts in Plp1+ extrastriolar SCs. As we can see in Figure 4, nearly half of the most abundantly expressed genes in Plp1+ cells were lowly or not expressed in Lgr5+ cells, but nearly all of the most highly expressed genes in Lgr5+ cells were also highly expressed (among the top 2000 expressed genes) in Plp1+ cells. These results indicate that Lgr5+ cells not only share the same characteristics as Plp1+ cells, but they also have their own unique characteristics. That is to say, the Lgr5+ cells appear to be a specialized subset of Plp1+ cells.

**Figure 5:**
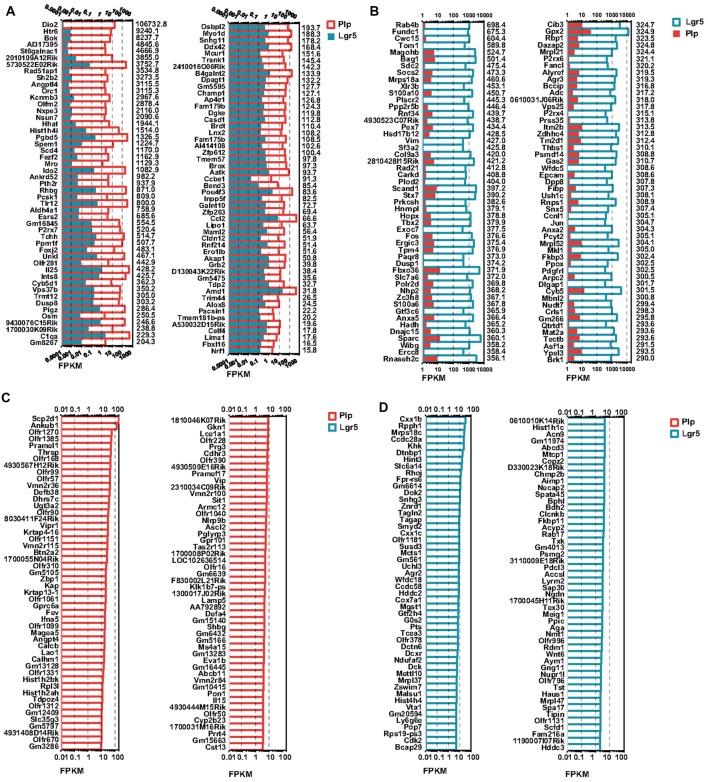
Differentially and uniquely expressed genes in Lgr5+ SCs and Plp1+ SCs. **(A)** The top 100 most differentially expressed genes in Plp1+ SCs. The numerical values on the right side of each panel represent the fold difference in expression for Plp1+ SCs vs. Lgr5+ SCs. **(B)** The top 100 most differentially expressed genes in Lgr5+ SCs. The numerical values on the right side of each panel represent the fold difference in expression for Lgr5+ SCs vs. Plp1+ SCs. **(C)** The 100 uniquely expressed genes in Plp1+ SCs. The numerical values on the right side of each panel represent the fold difference in expression for Plp1+ SCs vs. Lgr5+ SCs. **(D)** The 100 uniquely expressed genes in Lgr5+ SCs. The numerical values on the right side of each panel represent the fold difference in expression for Lgr5+ SCs vs. Plp1+ SCs. The length of the bar after each gene represents its FPKM value.

To determine which genes are differentially expressed in Lgr5+ striolar SCs and Plp1+ extrastriolar SCs, we compared the expression levels of all of the transcripts in Lgr5+ striolar SCs and Plp1+ extrastriolar SCs. Differentially expressed genes were categorized as either genes whose expression levels were at least above 1.5-fold different between the two groups or genes that were uniquely expressed in one or the other subset. Figures 5A,B show the top 100 differentially expressed genes and Figures 5C,D shows top 100 uniquely expressed genes in the two SC populations. Some of these differently and uniquely expressed genes have been reported previously, and they are involved in a variety of biological processes in the inner ear such as HC development, maintaining auditory processes, neuronal regeneration, etc., including some that were upregulated in Plp1+ SCs, including *Dio2* (Wangemann et al., 2009), *Meox2* (Lin et al., 2003), *kcnmb3* (Pyott and Duncan, 2016), *P2rx7* (Verderio et al., 2006), *C1qa* (Calton et al., 2014), *Osbpl2* (Thoenes et al., 2015; Xing et al., 2015), *Myo1d* (Dumont et al., 2002), *Ccl2* (Tan et al., 2016), and *Cldn12* (Matsubara et al., 2012), and some that were upregulated in Lgr5+ SCs, including *Vim* (Baier et al., 2017), *Col9a3* (Asamura et al., 2005), *Tbx2* (Hu et al., 2006), *Fos* (Suh et al., 2016), *Thbs1* (Mendus et al., 2014), and *Mbnl2* (Alvarado et al., 2009). In addition, *Vipr1* (Feng and Liu, 2015), *Ascl2* (Oh-McGinnis et al., 2011), and *Pon1* (Teranishi et al., 2012) were uniquely expressed in Plp1+ SCs, and *Agr2* (Tang et al., 2014) and *Wnt6* (Schubert et al., 2002) were uniquely expressed in Lgr5+ SCs. However, many of the differentially and uniquely expressed genes have not been described before in the inner ear, and further studies are needed.

### Cell Cycle, Transcription Factor and Signaling Pathway Analysis

**Figure 6:**
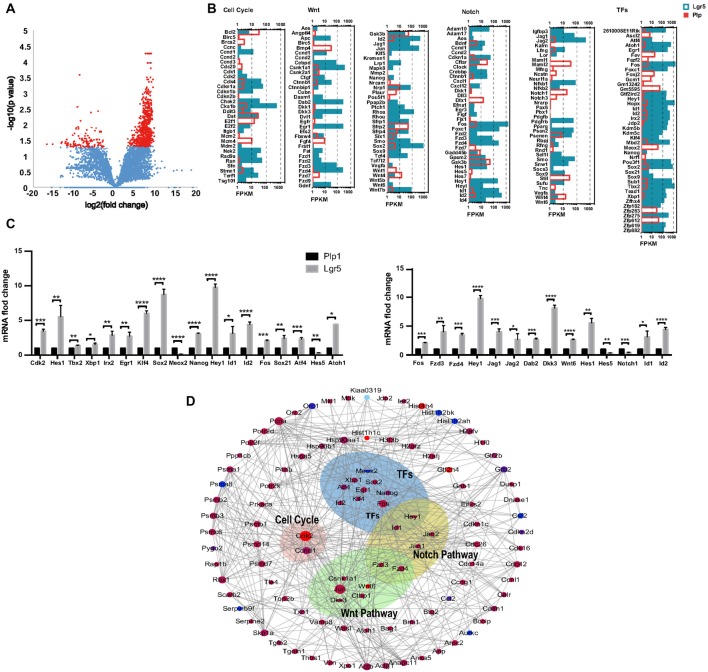
Wnt and Notch signaling pathway gene expression and cooperation between Lgr5+ SCs and Plp1+ SCs. **(A)** The volcano plot reflects the overall gene expression. The orange dots represent the differentially expressed genes, and the blue dots represent the non-differentially expressed genes. **(B)** The expression levels of genes involved in cell cycle regulation, the Wnt signaling pathway, the Notch signaling pathway, and transcription factors (TFs). The length of the bar after each gene represents its FPKM value. **(C)** Real-time PCR-validated differentially expressed genes involved in the cell cycle, TFs, and the Wnt and Notch signaling pathways. *p < 0.05, **p < 0.01, ***p < 0.001, ****p < 0.0001. **(D)** Gene interaction analysis of differentially expressed genes in Lgr5+ SCs (red) and Plp+ SCs (blue).

Lgr5+ SCs and Plp1+ SCs respond differently to the Notch and Wnt pathways and show different abilities for proliferation and differentiation. To determine which signaling pathway factors are involved in regulating the proliferation and HC regeneration ability of the two cell groups, we measured the transcription of genes involved in the Wnt and Notch pathways, cell cycle genes, and transcription factor (TF) genes (Figures 6A,B). We found that two cell cycle genes, *Cdk2* and *Ccnd1*, were significantly upregulated in Lgr5+ SCs. Among the TFs, *Atoh1*, *Hes1*, *Atf4*, *Sox21*, *Fos*, *Id1*, *Id2*, *Hey1*, *Nanog*, *Sox2*, *Klf4*, *Egr1*, *Irx2*, *Xbp1* and *Tbx2* were upregulated in Lgr5+ SCs, while *Meox2* was upregulated in Plp1+ SCs. Among the Wnt signaling pathway factors examined, *Wnt6*, *Fos*, *Fzd3*, *Fzd4* and *Dkk3* were significantly upregulated in Lgr5+ SCs compared to Plp1+ SCs. Among the Notch signaling factor genes, *Hes1*, *Jag1*, *Jag2*, *Hey1*, *Id1* and *Id2* were significantly upregulated in Lgr5+ SCs, while *Hes5* and *Notch1* were significantly upregulated in Plp1+ SCs. Most of the differentially expressed genes, including *Hes1* (Zheng et al., 2000), *Atoh1* (Bermingham et al., 1999; Lee et al., 2017), and *Jag1* (Hao et al., 2012), have already been reported in the inner ear. *Fzd1–4* have been reported to be expressed in SCs according to RNA-Seq analysis (Zhang et al., 2017), but their roles in the inner ear need to be further investigated. These results were confirmed by qPCR validation (Figure 6C).

In order to get a comprehensive perspective of the gene networks involved in utricular HC regeneration, we performed a STRING protein-protein interaction analysis, which assembles the predicted networks of the differentially expressed genes (fold change >2.0, *p* < 0.05) according to the functional categories highlighted by gene ontology analysis (Figure 6D). This analysis suggests that complex gene regulation patterns are involved in HC regeneration, including Wnt and Notch signaling pathway factors and other TFs.

## Discussion

It has been reported that the SCs of the mammalian utricle have a limited capacity for HC regeneration in response to HC damage (Li et al., 2003), and the striolar region is generally recognized as the most conducive area for HC regeneration. Therefore, determining the differences between SCs in the striolar and extrastriolar region might provide critical clues for the development of new strategies for HC regeneration. Here, we have isolated Lgr5-EGFP+ and Plp1-tdTomato+ SCs from transgenic mice by flow cytometry.

### Lgr5+ Cells Have Greater Capacity for Cell Proliferation and HC Differentiation

In this work we show for the first time a way to isolate striolar (Lgr5+) SCs and extrastriolar (Plp1+) SCs, which allows us to compare the differences in the SCs from the two regions of the utricles after neomycin damage. Although the Lgr5+ SCs are a smaller population than Plp1+ SCs, our results indicated that the majority of the spheres that gave rise to HCs originated from Lgr5+ SCs.

The sphere-forming assay and the differentiation assay showed that Lgr5+ striolar SCs have much higher capacity for proliferation and HC regeneration *in vitro* than Plp+ extrastriolar SCs. The increased yield of HCs from the spheres derived from Lgr5+ SCs in the utricle is consistent with the observation that Lgr5+ SCs are a population of progenitor cells in the cochlea (Shi et al., 2012). There are several hypotheses for this observed difference. First, *Lgr5* is regarded as a stem cell marker gene in many systems (Barker et al., 2007; Chai et al., 2012; Shi et al., 2012; Huch et al., 2013), so it is reasonable to hypothesize that these Lgr5+ SCs in the striolar region of the utricle are true stem cells. Second, The SCs in the striolar region are more immature compared to SCs in the extrastriolar region, and thus it is reasonable to hypothesize that these cells are not as fully differentiated as the extrastriolar cells. Third, Plp1 is not only expressed in extrastriolar SCs, but is also expressed in a portion of the glial cells and Schwann cells below the sensory epithelium in the utricle, and Plp1+ cells can differentiate not only into HCs, but also into neural cells. Thus, Lgr5+ cells are more specific for differentiation into HCs, while Plp+ cells are not able to fully differentiate into HCs because they need to retain the ability to differentiate into a broader array of cell types (McLean et al., 2016). A previous report showed that without Wnt stimulation Lgr5-GFP expression, as a marker of stem cells, was gradually diminished during *in vitro* culture, while Wnt activation could maintain the Lgr5-GFP expression during culture (McLean et al., 2017). Consistent with this previous report, we also found that Lgr5-GFP expression gradually disappeared during culture, especially in the cell differentiation culture, suggesting that the stemness of the Lgr5+ cells might gradually diminish in culture.

### Lgr5+ Cells Are More Responsive to the Notch and Wnt Signaling Pathways

The Notch and Wnt signaling pathways are highly conserved in mammals and non-mammalian vertebrates. In our previous study, we demonstrated that using the γ-secretase inhibitor DAPT to block Notch signaling removes the brakes on the canonical Wnt signaling pathway and thus induces Lgr5+ progenitor cells to mitotically generate HCs in the cochlea (Li et al., 2015). Canonical Wnt signaling is activated after tissue damage and mediates repair in the intestine, liver, bone, and inner ear (Wang et al., 2015). Lgr5, a known stem cell marker and a target of the Wnt pathway, can be induced by HC loss and lead to the regeneration of HCs by SCs as has been reported in our previous work (Wang et al., 2015).

The Wnt and Notch signaling pathways usually interact to regulate various biological reactions in a variety of tissues. In the developing cochlea, Wnt activity regulates HC formation and thus acts upstream of ATOH1, while Notch-mediated lateral inhibition prevents the SCs from adopting the HC fate by inhibiting ATOH1 expression. One of our recent publications shows that Notch signaling is an upstream and negative regulator of Wnt signaling during cochlear HC regeneration (Li et al., 2015). Similarly, in utricles the combination of Notch inhibition and Wnt activation can significantly promote SC proliferation and increase the number of regenerated HCs (Wu et al., 2016).

In the current study, we found that with either DAPT or BIO there was greater SC proliferation and HC mitotic regeneration in the striolar region than the extrastriolar region in the utricle, which is consistent with the results of our previous study (Wu et al., 2016). This indicated that there is a clear difference in signaling pathway response between SCs in the striolar and extrastriolar regions. Lgr5 itself acts as a receptor for R-spondins, which are potent Wnt signal enhancers, making Lgr5+ SCs more responsive to Wnt signaling pathways (Carmon et al., 2011; Chai et al., 2011; de Lau et al., 2011). Therefore, it is reasonable to argue that the striolar region in the utricle where Lgr5+ SCs are mainly located will be more responsive to the activation of the Wnt signaling pathway. The interaction between the Notch and Wnt signaling pathways or the Notch signaling pathway on its own might make the SCs in these two regions respond differently to the Notch and Wnt signaling pathways.

### Differentially Expressed Genes in Lgr5+ SCs and Plp1+ SCs

We found 283 genes that were differentially highly expressed in Lgr5+ SCs and 959 genes that were differentially highly expressed in Plp1+ SCs. Some of these genes were previously reported to be present in the inner ear and were highly expressed in Plp1+ SCs. *Dio2* and *Cldn12* are essential for the development and maturation of the inner ear (Wangemann et al., 2009). *Meox2* (also known as *Gax*) was reported to inhibit cellular proliferation, and down-regulation this gene might induce HC regeneration (Lin et al., 2003; Tan et al., 2016).

The set of previously reported genes among those that were highly expressed in Lgr5+ SCs includes *Vim*, *Col9a3*, *Tbx2*, *Agr2*, *Fos*, *Thbs1*, *Mbnl2* and *Wnt6*. *Vim* was reported to be expressed in two types of SCs in the cochlea—Deiters’ cells and inner pillar cells (Oesterle et al., 1990), and the expression pattern partly overlapped with the expression of *Lgr5* in the cochlea. The up-regulation of *Vim* in our sequencing results indicated that Lgr5+ cells in the utricle share similar characteristics with Lgr5+ cells in the cochlea. These results indicate that Lgr5+ SCs in the striolar region might be classified as a subset of SCs that have greater progenitor capacity in the utricle. High expression of *Agr2* has been identified in several human cancer cells, and it is considered to be a pro-oncogenic factor (Chevet et al., 2013).

We analyzed the cell cycle genes, TF genes and Notch/Wnt signal pathway factor genes that might be involved in cell proliferation and/or HC regeneration. We constructed a STRING prediction map that established a complex network of gene interactions, and these interactions might represent new therapeutic targets for HC regeneration.

#### Cell Cycle Analysis

The highly expressed genes that were only seen in Lgr5+ SCs include *Cdk2* and *Ccnd1*. *Ccnd1*, which encodes the protein Cyclin D1, acts as a cell cycle gene and is a Wnt or Notch target gene. Cyclin D1 is critical for the proliferative plasticity of SCs (Laine et al., 2010), and targeting the induction of *Ccnd1* in SCs promotes proliferative regeneration in the inner ear (Laine et al., 2010). The Cdk gene family encodes cyclin-dependent kinases (CDKs) that are involved in HC differentiation and SC proliferation (Malgrange et al., 2003). In a study of vestibular schwannoma patients, CDK2 was primarily localized in the vestibular nerve, and Schwann cells, axons, and ganglion cells had high CDK2 expression (Lasak et al., 2002), which indicates that CDK2 is related to the slow growth rate of these tumor. We also found other cell cycle genes, including *Dst* and so on, that were abundantly expressed in both cell populations, but the functions of these genes are not fully understood.

#### Transcription Factor Analysis

The highly expressed TF genes in Lgr5+ SCs include *Tbx2*, *Xbp1*, *Irx2*, *Egr1*, *Klf4*, *Sox2*, *Nanog*, *Hey1*, *Id1*, *Id2*, *Fos*, *Sox21* and *Atf4*. *Egr1* and c-*Fos* are immediate early genes, increasing rapidly and transiently in response to membrane potential changes or receptor coupled intracellular signals that have been shown to be involved in growth and remodeling in the peripheral auditory system and the brain (Sato et al., 1997). *Atf4*, which encodes activating TF 4, has been reported to be upregulated in the cochlear lateral wall in the presence of primary injuries (Fujinami et al., 2010) and to act as an endoplasmic reticulum (ER) stress marker (Fujinami et al., 2012). *Xbp1* is another ER stress-related gene, and Oishi et al. (2015) reported that aminoglycoside-induced ER stress and cell death in spiral ganglion neurons is mitigated by XBP1, which masks aminoglycoside neurotoxicity at the organism level. The increased levels of *Atf4* and *Xbp1* indicated that the Lgr5+ cells might play critical roles in the response to neomycin-induced HC damage (Oishi et al., 2015).

*Klf4*, *Sox2* and *Nanog* are stemness markers (Burns et al., 2012b; Lou et al., 2013), while *Tbx2* is an otic or early inner ear marker (Hu et al., 2006). Sox21 is normally expressed by sensory progenitors within the vestibular and auditory regions (Freeman and Daudet, 2012). The Iroquois (*Iro*/*Irx*) genes encode a family of homeodomain-containing TFs that possibly contribute to the neurogenic processes involved in forming the acoustic and vestibular ganglia (Cardeña-Núñez et al., 2017).

*Atoh1*, a member of the basic helix-loop-helix TF family, is well known to be required for HC regeneration (Zheng and Gao, 2000). The Id genes encode inhibitors of differentiation and DNA binding proteins, including *Id1*, *Id2* and *Id3*, and they have been shown to negatively regulate many basic helix-loop-helix TFs. In the cochlea, Ids are expressed within the cochlear duct in a pattern that is consistent with a role in the regulation of HC development (Jones et al., 2006). In the utricle, ID1, ID4 and ID2 all show a strong positive correlation with ATOH1 expression during utricular sensory epithelia regeneration (Ku et al., 2014). Therefore, the increased expression levels of *Id1* and *Id2* in Lgr5+ cells indicate the capacity of HC regeneration and are in accordance with *Atoh1* expression in Lgr5+ progenitors.

*Hey1* is a Notch target gene. In studies of HC-damaged cochlear cultures, *Hey1* expression was only modestly reduced, while it was significantly down-regulated in the presence of DAPT and gentamicin *in vitro*. This indicated that *Hey1* expression is maintained by a Notch-dependent mechanism (Korrapati et al., 2013).

The highly expressed TF genes in Plp1+ SCs include *Meox2* and *Ebf1*. *Ebf1* has not been studied in the inner ear, but *Ebf1* is up-regulated in the developing otocyst, suggesting tissue- and/or stage-specific activity of *Ebf1* (Sajan et al., 2011).

#### Wnt Signaling Pathway Analysis

The Wnt signaling factor genes that were highly expressed in Lgr5+ SCs include *Jun (c-jun)*, *Fzd3*, *Fzd4*, *Wnt6*, *Dkk3*, *Id2*, *Nanog* and *Sox2*, while few Wnt signaling factor genes were found in Plp1+ cells. *c-Jun* plays important roles in regulating cell cycle entry, cell proliferation and cell differentiation because it can remove p53-mediated inhibition of cell cycle entry (Shaulian et al., 2000). The increased level of *c-Jun* is likely, therefore, to play a role in the high proliferation level of Lgr5+ SCs observed in our study. The Fzd gene family members, which are Wnt receptor genes in mammals, have been implicated in a variety of developmental processes. *Fz3* is localized to a series of remarkably discrete zones both in HCs and SCs (Wang et al., 2006). *Fz4*, the only Frizzled receptor that is transcribed sporadically in the nonsensory lagena (Sienknecht and Fekete, 2008), is required for the maintenance of the vasculature within the cochlea (Xu et al., 2004). *Wnt6* is expressed in stem cells (Katoh, 2008) and is expressed along the nonsensory cochlear duct overlapping the nonsensory lagenar domain (Sienknecht and Fekete, 2008). The overlapping of *Wnt6* and *Fzd4* might mean that there is a relationship between these two Wnt-signaling genes and that they might be involved in the increased proliferation capacity of Lgr5+ SCs compared to Plp1+ SCs. Unlike other members of the Dickkopf family (*Dkk1*, *Dkk2* and *Dkk4*), which inhibit Wnt signaling, *Dkk-3* might activate Wnt signaling (Forsdahl et al., 2014). The precise mechanism by which *Dkk-3* is involved in Wnt signaling remains unclear, and further studies are required to answer this question.

#### Notch Signal Pathway Analysis

Among the Notch signaling pathway genes, *Hes1*, *Jag1*, *Jag2*, *Hey1*, *Id1* and *Id2* were significantly upregulated in Lgr5+ SCs, while *Hes5* and *Notch1* were upregulated in Plp1+ SCs. Notch1 is a receptor protein and Jag1 and Jag2 are ligands of the Notch signaling pathway (Kiernan et al., 2005). Jag1 and Jag2 are restricted to cells that will develop into HCs, and they are required for sensory progenitor development in mammals (Petrovic et al., 2014). Notch1 is also expressed in cells that will develop into SCs (Lanford et al., 1999). *Hes1* and *Hes5* are downstream genes of the Notch signaling pathway (Zheng et al., 2000; Zine et al., 2001). Examination of cochleae from *Hes1*^−/−^ and *Hes5*^−/−^ mice showed a significant increase in the number of HCs (Zine et al., 2001). In our work, *Hes1* was highly expressed in the Lgr5+ SCs, while *Hes5* was highly expressed in Plp1+ SCs, and the different expression patterns in the two subsets of SCs indicate that *Hes1* and *Hes5* might be involved in different mechanisms of cell fate determination via the Notch signaling pathway.

In summary, we have established an experimental model to identify and distinguish between the striolar and extrastriolar SCs in neonatal mouse utricles after neomycin damage. These two subsets of SCs are involved in different proliferation and differentiation mechanisms, as well as in different responses to Notch and Wnt signaling. The analysis of the differentially expressed genes of these two subsets of SCs can contribute to a better understanding of the possible mechanisms behind cell proliferation and differentiation and might suggest potential therapeutic targets for HC regeneration in the inner ear.

## Author Contributions

DY, WL and SS performed the experiment. LG and YC analyzed the data. SS, RC and HL conceived the idea. SS wrote the manuscript.

## Conflict of Interest Statement

The authors declare that the research was conducted in the absence of any commercial or financial relationships that could be construed as a potential conflict of interest.

## Supplementary Material

FIGURE S1 Sphere-forming ability assay of Lgr5+ and Plp1+ supporting cells (SCs). **(A)** Sorted Lgr5-EGFP+ and Plp1-tdTomato+ cells were cultured separately. EGFP+ cells were observed in neurospheres derived from Lgr5+ progenitors **(Aa,b)**, while tdTomato+ cells were derived from Plp1+ colonies **(Ac,d)**. **(B)** Spheres from the first two generations from Lgr5-EGFP+ progenitors and Plp1-tdTomato+ cells **(Ba–d)**. **(C)** Quantification of sphere numbers **(Ca)** and sizes **(Cb)** after multiple passages from Lgr5+ progenitors or Plp1+ SCs. Data are shown as the mean ± SD. Two-way ANOVA, ****p* < 0.001, *n* = 4 in **(Ba)** and *n* = 10 in **(Bb)**. **(D,E)** Differentiation assay of the first-generation spheres from Lgr5+ progenitors and Plp1+ SCs. A high percentage of Myosin7a+ hair cells (HCs) was observed inside the colonies generated from Lgr5+ SCs **(Da–d)**, and a portion of the differentiated cells were EdU+ (**Db**, arrowheads), but a low percentage of Myosin7a+ HCs was identified by differentiation of Plp1+ clones **(Ea–d)**. **(F)** Quantification of the percentage of Myosin7a+ cells **(Fa)** and Myosin7a+/EdU+ **(Fb)** cells derived from Lgr5+ spheres and Plp1+ spheres. Data are shown as the mean ± SD. *t*-test, ****p* < 0.001, *****p* < 0.0001. *n* = 4. Scale bars are 50 μm in **(B)** and 20 μm in **(A,D,E)**.

FIGURE S2 Differentiation capacity of Lgr5+ and Plp1+ SCs. **(A)** Schematic illustration of the experimental protocol for HC regeneration by sorted Lgr5+ and Plp1+ SCs cultured with EdU *in vitro*. **(Ba–f)** Lgr5+ and **(Ca–f)** Plp1+ SCs generated Myosin7a+ cells *in vitro*. **(D)** Quantification of the numbers of colonies and cells formed from Lgr5+ or Plp1+ SCs. **(Da)** The total colonies and Myosin7a+ colonies formed from Lgr5+ or Plp1+ SCs. **(b)** The total number of Myosin7a+ cells and the numbers of Myosin7a+ cells inside or outside of the colony formed from Lgr5+ or Plp1+ SCs. **(c)** The numbers of Myosin7a+/EdU+ cells inside or outside of the colonies formed by Lgr5+ or Plp1+ SCs. Data are shown as the mean ± SD. ***p* < 0.01, ****p* < 0.001, *n* = 3. Scale bars are 20 μm in **(B,C)**.

TABLE S1 Differentiation assay after sphere forming for Lg5+ and Plp1+ SCs.

TABLE S2 Primers used for mouse genotyping.

TABLE S3 Primers for qPCR validation.
