# Supplementary figures and images for: Characterization of Wnt and Notch-Responsive Lgr5+ Hair Cell Progenitors in the Striolar Region of the Neonatal Mouse Utricle
Source: Front Mol Neurosci. 2018 Apr 30;11:137. doi: 10.3389/fnmol.2018.00137 (PMC5937014; doi:10.3389/fnmol.2018.00137)

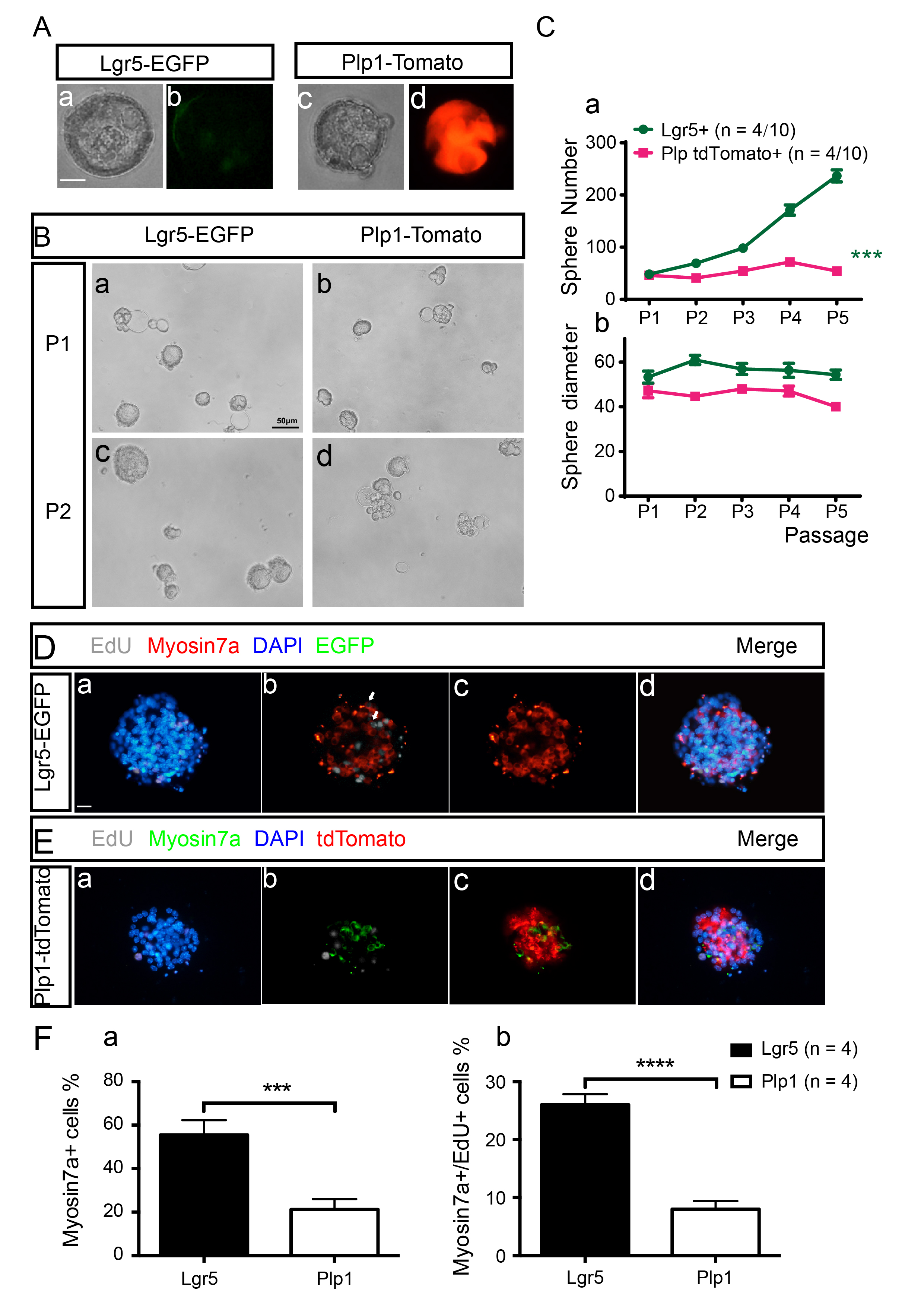

Supplement: FIGURE S1 — Sphere-forming ability assay of Lgr5+ and Plp1+ supporting cells (SCs). (A) Sorted Lgr5-EGFP+ and Plp1-tdTomato+ cells were cultured separately. EGFP+ cells were observed in neurospheres derived from Lgr5+ progenitors (Aa,b), while tdTomato+ cells were derived from Plp1+ colonies (Ac,d). (B) Spheres from the first two generations from Lgr5-EGFP+ progenitors and Plp1-tdTomato+ cells (Ba–d). (C) Quantification of sphere numbers (Ca) and sizes (Cb) after multiple passages from Lgr5+ progenitors or Plp1+ SCs. Data are shown as the mean ± SD. Two-way ANOVA, ***p < 0.001, n = 4 in (Ba) and n = 10 in (Bb). (D,E) Differentiation assay of the first-generation spheres from Lgr5+ progenitors and Plp1+ SCs. A high percentage of Myosin7a+ hair cells (HCs) was observed inside the colonies generated from Lgr5+ SCs (Da–d), and a portion of the differentiated cells were EdU+ (Db, arrowheads), but a low percentage of Myosin7a+ HCs was identified by differentiation of Plp1+ clones (Ea–d). (F) Quantification of the percentage of Myosin7a+ cells (Fa) and Myosin7a+/EdU+ (Fb) cells derived from Lgr5+ spheres and Plp1+ spheres. Data are shown as the mean ± SD. t-test, ***p < 0.001, ****p < 0.0001. n = 4. Scale bars are 50 μm in (B) and 20 μm in (A,D,E). [file Image_1.JPEG]

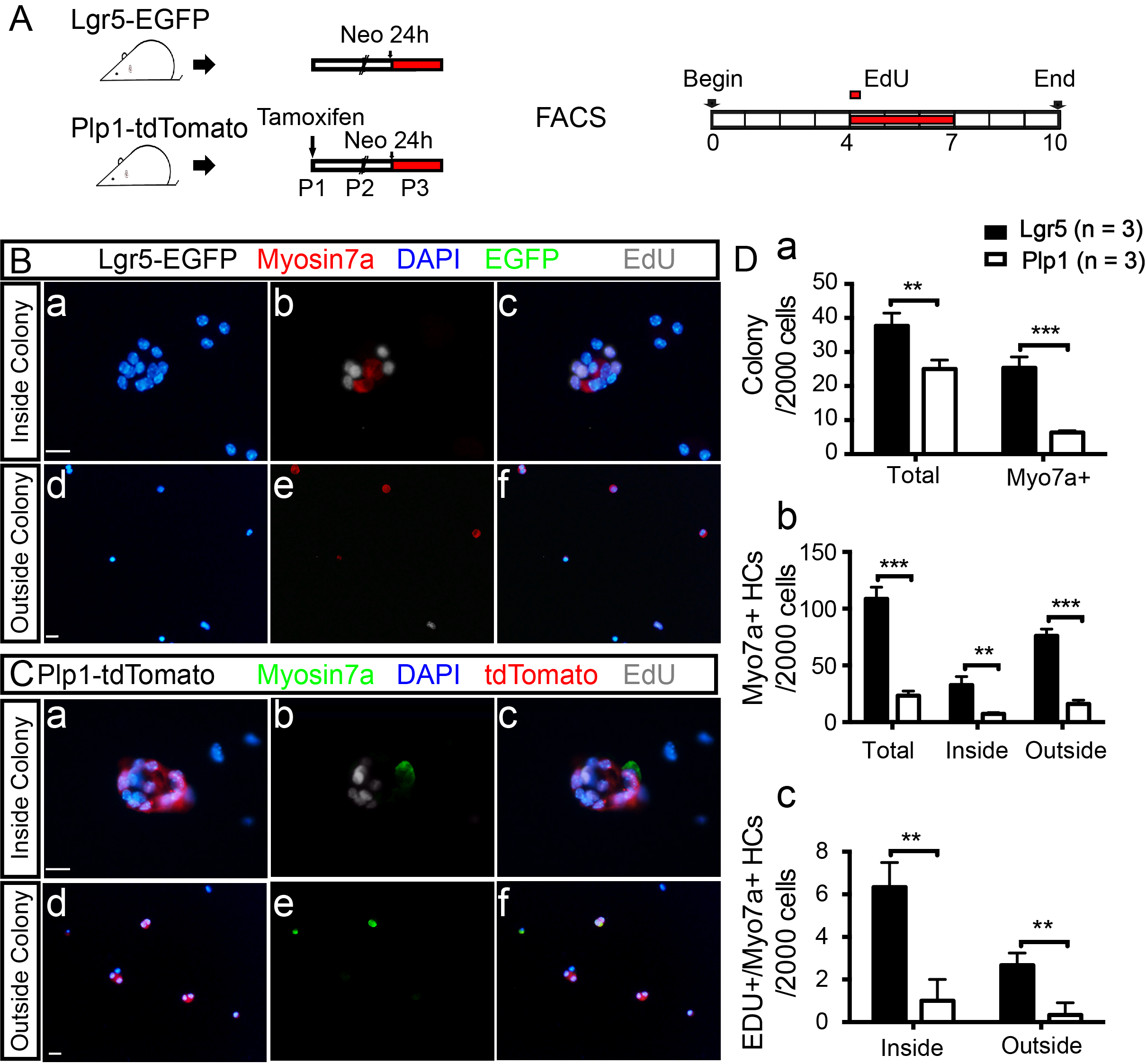

Supplement: FIGURE S2 — Differentiation capacity of Lgr5+ and Plp1+ SCs. (A) Schematic illustration of the experimental protocol for HC regeneration by sorted Lgr5+ and Plp1+ SCs cultured with EdU in vitro. (Ba–f) Lgr5+ and (Ca–f) Plp1+ SCs generated Myosin7a+ cells in vitro. (D) Quantification of the numbers of colonies and cells formed from Lgr5+ or Plp1+ SCs. (Da) The total colonies and Myosin7a+ colonies formed from Lgr5+ or Plp1+ SCs. (b) The total number of Myosin7a+ cells and the numbers of Myosin7a+ cells inside or outside of the colony formed from Lgr5+ or Plp1+ SCs. (c) The numbers of Myosin7a+/EdU+ cells inside or outside of the colonies formed by Lgr5+ or Plp1+ SCs. Data are shown as the mean ± SD. **p < 0.01, ***p < 0.001, n = 3. Scale bars are 20 μm in (B,C). [file Image_2.JPEG]
