# Supplementary material for: Characterization of Wnt and Notch-Responsive Lgr5+ Hair Cell Progenitors in the Striolar Region of the Neonatal Mouse Utricle
Source: Front Mol Neurosci. 2018 Apr 30;11:137. doi: 10.3389/fnmol.2018.00137 (PMC5937014; doi:10.3389/fnmol.2018.00137)
Supplement: TABLE S1 — Differentiation assay after sphere forming for Lg5+ and Plp1+ SCs. [file Table_1.DOCX]

**Table S1: Differentiation assay after sphere forming for Lg5+ and Plp1+ SCs.**

|  | Lgr5-EGFP | | | Plp-tdTomato | | |
| --- | --- | --- | --- | --- | --- | --- |
|  | Myo7a+ | Myo7a+/EdU+ | Total | Myo7a+ | Myo7a+/EdU+ | Total |
| Sphere 1 | 47 | 12 | 82 | 7 | 1 | 47 |
| Sphere 2 | 54 | 15 | 116 | 10 | 2 | 80 |
| Sphere 3 | 64 | 17 | 118 | 12 | 2 | 66 |
| Sphere 4 | 48 | 11 | 84 | 14 | 1 | 64 |
