# Supplementary material for: Characterization of Wnt and Notch-Responsive Lgr5+ Hair Cell Progenitors in the Striolar Region of the Neonatal Mouse Utricle
Source: Front Mol Neurosci. 2018 Apr 30;11:137. doi: 10.3389/fnmol.2018.00137 (PMC5937014; doi:10.3389/fnmol.2018.00137)
Supplement: TABLE S2 — Primers used for mouse genotyping. [file Table_2.DOCX]

**Table S2: Primers used for mouse genotyping.**

| **Gene** |  |  | **Primers** |
| --- | --- | --- | --- |
| Lgr5 | WT | **Forward** | 5′-ATACCCCATCCCTTT TGAGC-3′ |
|  |  | **Reverse** | 5′-CTGCTCTCTGCTCCCAGTCT-3′ |
|  | Mutant | **Forward** | 5′-GAACTTCAGGGTCAG CTTGC-3′ |
|  |  | **Reverse** | 5′-CTGCTCTCTGCTCCCAGTCT-3′ |
|  | | | |
| tdTomato | WT | **Forward** | 5′-CCGAAAATCTGTGGG AAGTC-3′ |
|  |  | **Reverse** | 5′-AAGGGAGCTGCAGTGGAGTA-3′ |
|  | Mutant | **Forward** | 5′-CTGTTCCTGTACGG CATGG-3′ |
|  |  | **Reverse** | 5′-GGCATTAAAGCAGCGTATCC-3′ |
|  | | | |
| Plp(Cre) | Cre | **Forward** | 5′-GCGGTCTGGCAGTAAAAACTATC-3′ |
|  |  | **Reverse** | 5′-GTGAAACAGCATTGCTGTCATT-3′ |
