# Supplementary material for: Characterization of Wnt and Notch-Responsive Lgr5+ Hair Cell Progenitors in the Striolar Region of the Neonatal Mouse Utricle
Source: Front Mol Neurosci. 2018 Apr 30;11:137. doi: 10.3389/fnmol.2018.00137 (PMC5937014; doi:10.3389/fnmol.2018.00137)
Supplement: TABLE S3 — Primers for qPCR validation. [file Table_3.DOCX]

**Table S3: Primers for qPCR validation.**

| **Gene** |  | **Primer** |
| --- | --- | --- |
| Gapdh | Forward | 5′-GCAAAGTGAGCAGCTACCAT-3′ |
|  | Reverse | 5'-GAAGGGGCGGAGATGATGA-3' |
| Hes1 | Forward | 5'-ACGACACCGGACAAACCA-3′ |
|  | Reverse | 5′-ATGCCGGGAGCTATCTTTCT-3′ |
| Hes5 | Forward | 5′-TGCTCAGTCCCAAGGAGAAA-3′ |
|  | Reverse | 5′-AGCTTGGAGTTGGGCTGGT-3′ |
| Atoh1 | Forward | 5'-GGGGTTGTAGTGGACGAGC-3' |
|  | Reverse | 5'-CGTTGTTGAAGGACGGGATAAC-3' |
| Dkk3 | Forward | 5'-CCATGTGCACCAGGAAGTTC-3' |
|  | Reverse | 5'-TCTTGCCTTCTTCATCCCCT-3' |
| Jag1 | Forward | 5′-TGTGCAAACATCACTTTCACCTTT-3′ |
|  | Reverse | 5′-GCAAATGTGTTCGGTGGTAAGAC-3′ |
| Jag2 | Forward | 5′-ACTGTGACTGTGAGACCAACT-3′ |
|  | Reverse | 5′-AGGTATTGGTCAGGCTCAGC-3′ |
| Fzd4 | Forward | 5'-TGACAACTTTCACGCCGC-3' |
|  | Reverse | 5'-CGATGGGGATGTTGATCTTCTC-3’ |
| Fzd3 | Forward | 5'-GCAAAGTGAGCAGCTACCAT-3' |
|  | Reverse | 5'-GTCTCTCCTCCATTCCTCGG-3' |
| Wnt6 | Forward | 5'-CAGTTCCAGTTCCGTTTCCG-3’ |
|  | Reverse | 5'-CGAAAGCTGTCTCTCGGATG-3' |
| Cdk2 | Forward | 5'-GCTCTCACGGGCATTCCT-3’ |
|  | Reverse | 5'-TAAGGTCTCGGTGAAGGACA-3’ |
| Fos | Forward | 5'-GAATCGGAGGAGGGAGCTG-3’ |
|  | Reverse | 5'-CAATCTCAGTCTGCAACGC-3’ |
| Dab2 | Forward | 5'-TCCCACCTCCACAAAGTACC-3’ |
|  | Reverse | 5'-CTGGAGGTTCTGAGGCAAAG-3’ |
| Atf4 | Forward | 5'-GCAGCAGCACCAGGCTCT-3' |
|  | Reverse | 5'-CAACACTGCTGCTGGATTTC-3’ |
| Egr1 | Forward | 5'-GAGCGAACAACCCTATGAGC-3' |
|  | Reverse | 5'-GGGATAACTCGTCTCCACCA-3’ |
| Irx2 | Forward | 5'-TTCCAGAAGCAAGGAGGAGA-3’ |
|  | Reverse | 5'-GTGAGCGAGTCGACGTGTAG-3’ |
| Klf4 | Forward | 5'-GGAGAAGACACTGCGTCCAG-3’ |
|  | Reverse | 5'-AGAGAGTTCCTCACGCCAAC-3' |
| Meox2 | Forward | 5'-AGAAGTGGCAGCAAAAGGAA-3' |
|  | Reverse | 5'-CCTTCCTAGGTTTGCTGTTCA-3' |
| Sox2 | Forward | 5'-GCGGAGTGGAAACTTTTGTC-3' |
|  | Reverse | 5'-CTCCGGGAAGCGTGTACTTA-3’ |
| Sox21 | Forward | 5'-GGGCTTCTCGGCTCAACT-3' |
|  | Reverse | 5'-TCCTCTGTCCCGTTGCTTT-3' |

| **Gene** |  | **Primer** |
| --- | --- | --- |
| Tbx2 | Forward | 5'-GATCGACAACAACCCCTTTG-3' |
|  | Reverse | 5'-CGGCTTACAGTGCTCCTCAT-3’ |
| Xbp1 | Forward | 5'-ATCCTGACGAGGTTCCAGAG-3’ |
|  | Reverse | 5'-GCTGCAGAGGTGCACATAGT-3’ |
| Nanog | Forward | 5'-GATGAAGTGCAAGCGGTGG-3’ |
|  | Reverse | 5'-GATGCGTTCACCAGATAGCC-3’ |
| Id1 | Forward | 5'-ACCCTGAACGGCGAGATC-3' |
|  | Reverse | 5'-CCTCAGCGACACAAGATGC-3' |
| Id2 | Forward | 5'-GAACACGGACAGCATCC-3' |
|  | Reverse | 5'-CCTGGTGAAATGGCTGATAACA-3' |
| Hey1 | Forward | 5'-CCAGTGCCTTTGAGAAGCAG-3' |
|  | Reverse | 5'-AGTGTGCAGCATTTTCAGGT-3' |
| Notch1 | Forward | 5'-CCCTCACCCTGCCAGAATG-3' |
|  | Reverse | 5'-TGCAGTTGTTTCCTGGACAG-3' |
